# The IQ‐compete assay for measuring mitochondrial protein import efficiencies in living yeast cells

**DOI:** 10.1002/1873-3468.70206

**Published:** 2025-10-25

**Authors:** Yasmin Hoffman, Annika Egeler, Saskia Rödl, Johannes M. Herrmann

**Affiliations:** ^1^ Cell Biology University of Kaiserslautern, RPTU Germany

**Keywords:** fluorescence quenching, genetically encoded sensors, mitochondria, presequences, protein targeting

## Abstract

Most mitochondrial proteins are synthesized in the cytosol and imported into the organelle. Here, we describe a novel Import and de‐Quenching Competition (IQ‐compete) assay which monitors the import efficiency of model proteins by fluorescence in living cells. For this method, the sequence of the tobacco etch virus (TEV) protease is fused to a mitochondrial precursor and coexpressed with a cytosolic reporter which becomes fluorescent upon TEV cleavage. Thus, inefficient import of the fusion protein leads to a fluorescent signal. With the IQ‐compete assay, the import efficiency of proteins can be reliably analyzed in fluorescence readers, by flow cytometry, by microscopy, and by western blotting. We are convinced that the IQ‐compete assay will be a powerful strategy for many different applications.

**Impact statement**

This article describes a novel method to monitor the mitochondrial import efficiency for a given protein in living yeast cells. With this IQ‐compete assay, protein import efficiencies can be quantified by fluorescent microscopy, flow cytometry, fluorescence spectrometry or western blotting.

## Graphical abstract

The efficiency of mitochondrial protein import depends on the properties of the newly synthesized precursor proteins. The Import and de‐Quenching Competition (IQ‐compete) assay is a novel method to monitor the import efficiency of different proteins by fluorescence in living yeast cells. Thus, mitochondrial import efficiencies can now be easily quantified in individual cells.

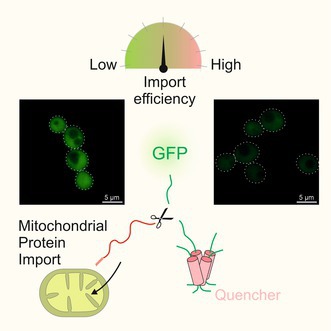

## Abbreviations

**DHFR**, dihydrofolate reductase

**ER**, endoplasmic reticulum

**HA**, hemagglutinine epitope

**IQ‐compete assay**, Import and de‐Quenching Competition assay

**MoClo**, modular cloning

**TCA**, tricarbonic acid

**TCS**, TEV cleavage site

**TEV**, tobacco etch virus protease

**TIM**, translocase of the inner membrane of mitochondria

**TOM**, translocase of the outer membrane of mitochondria

Intracellular membranes that define different types of compartments are a hallmark of eukaryotic cells. This compartmentalization makes it necessary that many newly synthesized proteins are translocated across membranes in order to reach different intracellular locations such as the endoplasmic reticulum (ER), mitochondria, peroxisomes, chloroplasts, or the nucleus. Targeting signals serve as address labels on these proteins and allow their reliable intracellular sorting [1, 2]. The combination of genetics and *in vitro* translocation reactions proved to serve as a very powerful strategy to identify the components of the different translocation machineries, as well as to study how they recognize their protein clients and thread them across the different intracellular membranes. The use of specific model proteins that were consistently used by many researchers in different laboratories allowed conclusions about the general principles of intracellular protein targeting that are now generally accepted textbook knowledge.

In the case of mitochondria, most precursor proteins are synthesized with N‐terminal matrix targeting signals that are both necessary and sufficient for mitochondrial protein import [3]. These presequences are typically removed upon protein translocation into the mitochondrial matrix by the matrix processing peptidase MPP [4, 5, 6]. The presequences bind to mitochondrial surface receptors and direct precursors through protein‐conduction channels of the translocases of the outer membrane (TOM) and the inner membrane (TIM23) complexes. The details of the translocation reactions were elucidated in depth and are described in excellent review articles [7, 8, 9, 10].

**Fig. 1:**
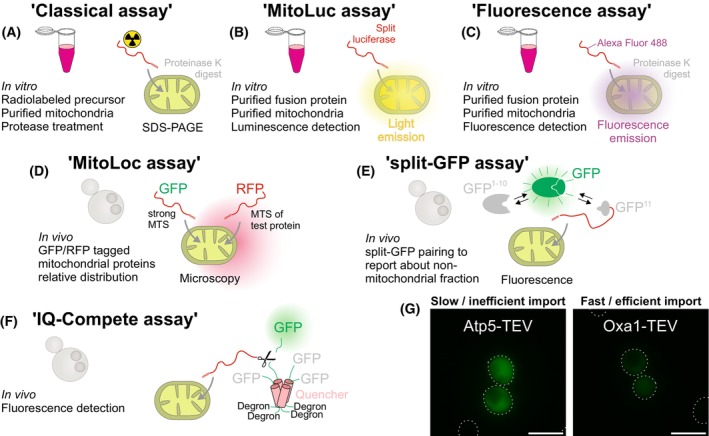
The import of proteins into mitochondria can be monitored with different assays. (A–C) Isolated mitochondria can be incubated with precursor proteins *in vitro*; these assays allow it to determine the kinetic parameters of the import reaction and are very well suited to dissect the molecular details of the protein translocation across mitochondrial membranes. (D–F) Schematic representation of *in vivo* assays which were developed to study the import reaction in living cells. GFP, green fluorescent protein; RFP, red fluorescent protein. (G) The IQ‐compete assay described in this study is a powerful approach to distinguish the transient cytosolic accumulation of slowly imported precursor proteins (Atp5) from the efficient import reaction driven by strong import signals (Oxa1). TEV, tobacco etch virus protease. Scale bars: 5 μm.

The mitochondrial protein import system was studied by an *in vitro* assay in which proteins are synthesized in reticulocyte lysate [11] and mixed with mitochondria purified from yeast cells [12]. Upon treatment with protease, the relative amounts of imported, protease‐inaccessible protein are monitored by SDS/PAGE and autoradiography (Fig. 1A). Proteaceous factors present in extracts of rabbit and rat cells were found to be particularly efficient in maintaining protein import competence *in vitro* [13, 14, 15]. However, their relevance under physiological import conditions could not always be confirmed and might be artifacts of the *in vitro* approach. Recent studies rather suggest that the cooperative binding of different factors, including the predominant chaperones of the cytosolic Hsp70 and Hsp90 systems, promotes protein translocation into mitochondria [16, 17, 18, 19, 20].

Some presequences were found to be particularly powerful and strongly import‐promoting. Examples of these “strong” matrix targeting sequences were used as preferred model proteins, including the first 69 residues of the subunit 9 of the ATP synthase complex of *Neurospora crassa* (Su9), a mutated matrix‐mistargeting version of the yeast cytochrome *b*_*2*_ (b_2_Δ19), or the inner membrane protein Oxa1 [12, 16, 21, 22, 23, 24, 25]. Presequences of other proteins were found to be much “weaker”; even though, in many cases, this lower import efficiency might be attributed to the fact that these proteins might be less compatible with the reticulocyte‐based *in vitro* import assay.

Several alternative import assays had been developed to overcome the limitations of the reticulocyte‐based approach: Pulse labeling with radioactivity followed by immunoprecipitation can measure the conversion of precursors to mature protein species *in vivo* [26, 27]. Moreover, a split luciferase reporter named MitoLuc can detect mitochondrial import by luminescence in real time (Fig. 1B) [25, 28] or mitochondrial precursors were labeled with fluorescent dyes such as Alexa Fluor 488 (Fig. 1C) [29]. However, in both cases, proteins must be recombinantly expressed and purified from bacteria first. Thus, these approaches are restricted to precursors which do not require the assistance of cytosolic chaperones. *In vivo* assays can overcome these problems: for example, in the comparative MitoLoc assay, the relative import efficiency of a strong (Su9) and a weak (Cox4) presequence is compared; this assay is useful to distinguish import‐competent from compromised mitochondria (Fig. 1D); however, it is not well suited to study the import signals in proteins, and it is technically demanding, as it requires monitoring the sublocalization of proteins within cells by confocal microscopy [30]. Alternatively, split‐GFP assays were used, for example, to show the accumulation of non‐imported proteins in the cytosol [31, 32, 33, 34]. While this assay can be read out by fluorescence intensity without the need of a microscope, it has the disadvantage that the tight association of the two split‐GFP parts can interfere with the intracellular location by artificial trapping of the protein (Fig. 1E). Thus, there is the need for further *in vivo* assays that are more reliable.

In this study, we describe a novel *in vivo* assay, called IQ‐compete (for Import and de‐Quenching Competition assay, Fig. 1F and G), which relies on the import‐dependent dequenching of a cytosolic GFP reporter [16]. This assay monitors the import efficiency of individual cells by fluorescence and can be used under different growth conditions. It is quantitative and can reliably distinguish weak from strong mitochondrial targeting signals. The details about how to use this assay are described in this article.

## Materials and methods

### Yeast strains and plasmids

Yeast strains used in this study are based on the wild type strains W303 (MATα *leu2‐3112 trp1‐1 can1‐100 ura3‐1 ade2‐1 his3‐11,15*) and BY4741 (MATa *his3Δ1 leu2Δ0 met15Δ0 ura3Δ0*).

For the generation of all constructs, the modular cloning (MoClo) toolkit [35] was used. The GFP‐Q‐D construct that was consistently used in this study consisted of the sequences of eGFP, the TEV cleavage site, the quencher sequence, and the CL1 degron. The sequence is provided in Table 2. This sequence was cloned into an expression plasmid under the control of the promoters listed in Table 1 as described previously [35]. For genomic integration, the sequence was fused to the hygromycin resistance cassette and integrated into the *YPRCΔ15* locus [36].

**Table 1:** Plasmids used in this study.

Identifier	Plasmid	Figure	Promotor
cHHYTK330	GFP‐TCS‐Quencher (GFP‐Q), *URA3*	2	*TEF1*
cHHYTK343	GFP‐TCS‐Quencher‐HA, *URA3*	2	*TEF1*
cHHYTK350	GFP‐TCS‐Quencher‐CL1 (GFP‐Q‐D), *URA3*	2	*TEF1*
cHHYTK351	GFP‐Q‐D, DHFR‐TEV, *URA3*	2, 4	*TEF1, TEF2*
cHHYTK352	GFP‐Q‐D, Atp5‐DHFR‐TEV, *URA3*	4	*TEF1, TEF2*
cHHYTK353	GFP‐Q‐D, Oxa1‐DHFR‐TEV, *URA3*	4	*TEF1, TEF2*
cHHYTK354	GFP‐Q‐D, DHFR‐TEV, *URA3*	4	*TEF1, ALD6*
cHHYTK355	GFP‐Q‐D, Atp5‐DHFR‐TEV, *URA3*	4	*TEF1, ALD6*
cHHYTK356	GFP‐Q‐D, Oxa1‐DHFR‐TEV, *URA3*	4	*TEF1, ALD6*
cHHYTK364	GFP‐Q‐D, integrated (*YPRCΔ15*)	2, 3, 4, 5, 6	*TEF1*
cHHYTK367	DHFR‐TEV, integrated (*YORWΔ22*)	2, 3, 4, 5	*TEF2*
cHHYTK368	Atp5‐DHFR‐TEV, integrated (*YORWΔ22*)	1, 3, 4, 5	*TEF2*
cHHYTK369	Oxa1‐DHFR‐TEV, integrated (*YORWΔ22*)	1, 3, 4, 5	*TEF2*
cHHYTK416	Atp5 (full length)‐TEV, integrated (*YORWΔ22*)	6	*TEF2*
cHHYTK417	Oxa1 (full length)‐TEV, integrated (*YORWΔ22*)	6	*TEF2*

For the different TEV‐containing constructs, the parts for the promoter regions, the presequences of Atp5 (residue 1–27) and Oxa1 (1–51), DHFR, uTEV3 [37], a terminator, and a kanamycin (G418) resistance cassette were combined by a MoClo strategy [35] as shown in Table 2. This sequence was integrated together into the *YORWΔ22* locus.

**Table 2:** Sequences of the reporter constructs used in this study. DHFR, dihydrofolate reductase; GFP, Green fluorescent protein; MTS, mitochondrial targeting signal; TCS, TEV cleavage site; TEV, tobacco etch virus protease.

**Sequence of the GFP‐Q‐D reporter (GFP‐TCS‐Quencher‐CL1)**
MSKGEELFTGVVPILVELDGDVNGHKFSVSGEGEGDATYGKLTLKFICTTGKLPVPWPTLVTTFTYGVQCFSRYPDHMKRHDFFKSAMPEGYVQERTIFFKDDGNYKTRAEVKFEGDTLVNRIELKGIDFKEDGNILGHKLEYNYNSHNVYIMADKQKNGIKVNFKIRHNIEDGSVQLADHYQQNTPIGDGPVLLPDNHYLSTQSALSKDPNEKRDHMVLLEFVTAAGITHGMDELYK GS ENLYFQS GA CNDSSDPLVVAASIIGILHLILWILDRL GSM ACKNWFSSLSHFVIHL *
The sequence consists of GFP, TEV cleavage site, Quencher, CL1 degron
**Sequence of the Oxa1** ^**MTS**^ **‐DHFR‐TEV construct**
MFKLTSRLVTSRFAASSRLATARTIVLPRPHPSWISFQAKRFNSTGPNANDVGS VRPLNCIVAVSQNMGIGKNGDLPWPPLRNEFKYFQRMTTTSSVEGKQNLVIMGRKTWFSIPEKNRPLKDRINIVLSRELKEPPRGAHFLAKSLDDALRLIEQPELASKVDMVWIVGGSSVYQEAMNQPGHLRLFVTRIMQEFESDTFFPEIDLGKYKLLPEYPGVLSEVQEEKGIKYKFEVYEKKD GSGESLFKGPRDYNPISSTICHLTNESDGHTTSLYGIGFGPFIITNKHLFRRNNGTLLVQSLHGVFKVKNTTTLQQHLIDGRDMIIIRMPKDFPPFPQKLKFREPQREERICLVTTNFQTKSMSSMVSDTSCTFPSSDGTFWKHWIQTKDGQCGNPLVSTRDGFIVGIHSASNFTNTNNYFASVPKNFMELLTNQEAQQWVSGWRLNADSVLWGGHKVFMVKPEEPFQPVKEATQLMNGS *
The sequence consists of MTS, DHFR, TEV
**Sequence of the Atp5** ^**MTS**^ **‐DHFR‐TEV construct**
MFNRVFTRSFASSLRAAASKAAAPPPV GS VRPLNCIVAVSQNMGIGKNGDLPWPPLRNEFKYFQRMTTTSSVEGKQNLVIMGRKTWFSIPEKNRPLKDRINIVLSRELKEPPRGAHFLAKSLDDALRLIEQPELASKVDMVWIVGGSSVYQEAMNQPGHLRLFVTRIMQEFESDTFFPEIDLGKYKLLPEYPGVLSEVQEEKGIKYKFEVYEKKD GSGESLFKGPRDYNPISSTICHLTNESDGHTTSLYGIGFGPFIITNKHLFRRNNGTLLVQSLHGVFKVKNTTTLQQHLIDGRDMIIIRMPKDFPPFPQKLKFREPQREERICLVTTNFQTKSMSSMVSDTSCTFPSSDGTFWKHWIQTKDGQCGNPLVSTRDGFIVGIHSASNFTNTNNYFASVPKNFMELLTNQEAQQWVSGWRLNADSVLWGGHKVFMVKPEEPFQPVKEATQLMNGS *
The sequence consists of MTS, DHFR, TEV

Yeast cells were grown at 25, 30, or 37 °C in yeast full medium containing 3% (w/v) yeast extract peptone (YP) broth (Formedium LTD, Swaffham, England) and 2% of the respective carbon source [glucose (D), galactose (Gal), lactate (L)]. Strains carrying plasmids were grown in minimal synthetic medium containing 0.67% (w/v) yeast nitrogen base and 2% of the respective carbon source. For plates, 2% of agar was added to the medium. All plasmids used in this study are listed in Table 1.

### Preparation of whole cell lysates

4 OD_600_ of yeast cells was harvested and washed with sterile water. Pellets were resuspended in 40 μL/OD_600_ Laemmli buffer containing 50 mm DTT. Cells were lysed using a FastPrep‐24 5 G homogenizer (MP Biomedicals, Irvine, CA, USA) with 3 cycles of 20 s, speed 6.0 m·s^−1^, 120 s breaks, glass beads (Ø 0.5 mm) at 4 °C. Lysates were heated at 96 °C for 5 min and stored at −20 °C.

### Antibodies

Antibodies against DHFR, GFP, Sod1, and Mia40 were raised in rabbits using recombinant purified proteins. The anti‐rabbit secondary antibody was obtained from Bio‐Rad (Hercules, CA, USA) [Goat Anti‐Rabbit IgG (H + L)–HRP Conjugate #172‐1019]. Antibodies were diluted in 5% (w/v) nonfat dry milk in 1x TBS buffer with the following dilutions: anti‐DHFR 1 : 500, anti‐GFP 1 : 500, anti‐Sod1 1 : 1000, anti‐Mia40 1 : 5000.

### Fluorescence microscopy

Cells were grown to mid‐log phase, and 1 OD_600_ was harvested via centrifugation. Cell pellets were resuspended in 30 μL PBS. 3 μL were pipetted onto a glass slide and covered with a cover slip. Manual microscopy was performed using a Leica Dmi8 Thunder Imager with an HC PL APO100×/1.44 Oil UV objective and Immersion Oil Type A 518 F. GFP was excited by light with a wavelength of 475 nm. Further processing of images was performed with the software las x (Leica Application Suite X version 3.7.5, Leica Microsystems, Wetzlar, Germany) and fiji/imagej.

### Fluorescence spectroscopy measurement in a plate reader

Cells were grown to mid‐log phase in SLac +0.2% glucose medium, and 4 OD_600_ of a yeast culture were harvested by centrifugation (5000 ***g***, 5 min). The pellet was resuspended in 400 μL SLac complete medium. Next, 100 μL (corresponding to 1 OD_600_) was transferred to a black flat‐bottomed 96‐well plate in technical triplicates. The plate was centrifuged at 30 ***g*** for 5 min to sediment the cells, and fluorescence (excitation 485 nm, emission 530 nm) was measured using a ClarioStar Fluorescence plate reader (BMG‐Labtech, Offenburg, Germany). Yeast cells not expressing any fluorescent protein were used for background subtraction. Each strain was measured in biological triplicates.

### Flow cytometry

For fluorescence intensity measurements, yeast cells were either grown to mid‐log phase in YPD, YPGal, or Lactate. 1 OD_600_ of cells were harvested, and cell pellets were resuspended in 500 μL PBS. The fluorescence intensity of 100 000 cells per strain was measured using the Attune™ Flow Cytometer (Thermo Fisher Scientific, Waltham, MA, USA). Each strain was measured in biological triplicates. Data analysis was performed using the flowjo™ software, version 10 (BD Life Sciences, Ashland, OR, USA).

### Miscellaneous

Protein extraction, western blotting, *in vitro* protein import assays, and image analysis were performed according to published procedures [16]. All raw data have been uploaded as Data S1.

## Results

### A cytosolic reporter can be efficiently controlled by protease‐triggered dequenching

**Fig. 2:**
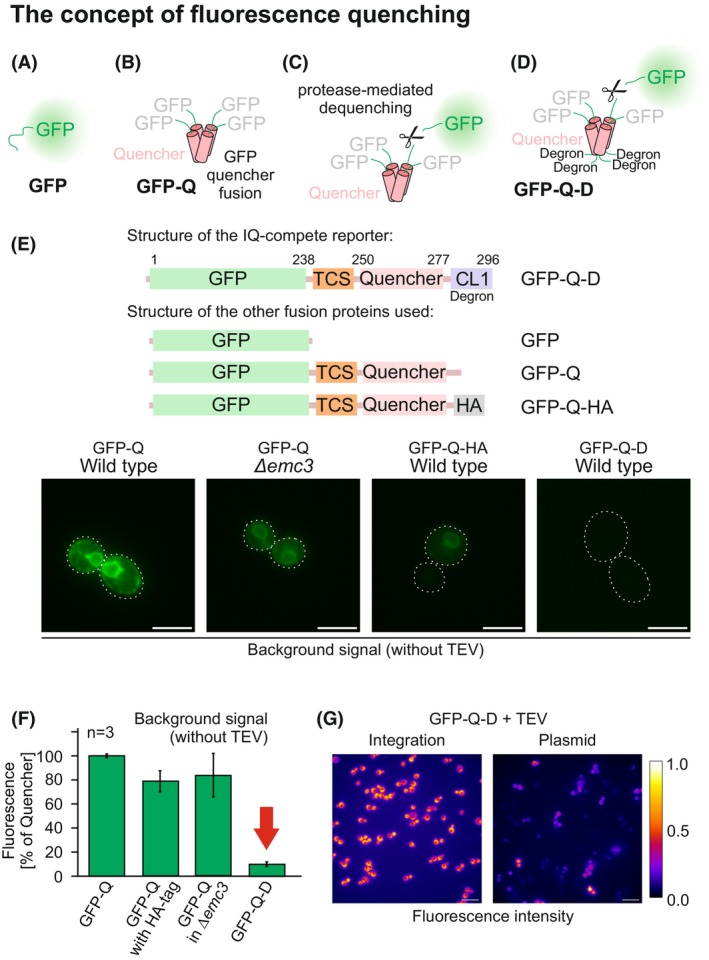
A quencher sequence fused to GFP efficiently abolishes fluorescence. (A–D) Schematic representation of the GFP‐quencher fusion proteins used in this study. GFP, green fluorescent protein; HA, hemagglutinin epitope. (E, F) The indicated fusion proteins were expressed in wild type or Δ*emc3* cells from a constitutive *TEF1* promoter. Cells were grown on SLac +0.2% glucose medium to mid‐log phase and analyzed by fluorescence microscopy or by fluorescence detection in a plate reader. See Table 2 for further information about the sequences of the fusion proteins. TCS, TEV cleavage site. Scale bar: 5 μm. (F) for quantification, mean values and standard deviations of three independent experiments are shown. The arrow highlights the minimal background fluorescence of the GFP‐Q‐D fusion protein. TEV, tobacco etch virus protease. (G) The genes for the GFP‐Q‐D fusion protein as well as for the cytosolic DHFR‐TEV fusion protein were placed on a single copy *CEN* plasmid or integrated into the genome before fluorescence was measured by microscopy. The signal intensity was visualized using the “Fire” lookup table in ImageJ to highlight intensity variations, with the color scale indicating normalized fluorescence intensities from 0.0 (black) to 1.0 (yellow/white). Scale bars represent 10 μm.

To develop an import reporter, we made use of a fluorescence dequenching strategy, based on the following hypothesis (Fig. 2A–D): The fluorescent signal of GFP (Fig. 2A) is abolished when GFP is fused to a 27‐residue long quencher sequence (GFP‐Q) [38]. This hydrophobic sequence tetramerizes and thereby prevents the maturation of the GFP chromophore (Fig. 2B). Proteolytic cleavage leads to GFP release and efficient dequenching, resulting in a ~ 50‐fold increase in fluorescence [38](Fig. 2C). This dequenching reporter was further improved by the addition of a C‐terminal CL1 degron sequence (GFP‐Q‐D) [39] to reduce the background fluorescence (Fig. 2D).

The addition of the degron sequence proved to be important (Fig. 2E). Initially, we had generated a fusion protein of GFP and the quencher linked by a sequence containing a tobacco etch virus protease cleavage site (TCS). However, expression of this GFP‐Q fusion resulted in considerable background fluorescence even in the absence of the TEV protease. This background staining showed the characteristic perinuclear staining of an ER localization (Fig. 2E, left panel) and is presumably caused by the insertion of the C‐terminal quencher region into the ER membrane. The quencher sequence forms a hydrophobic helix and thus mimics a C‐terminal tail anchor signal [40, 41]. Mutants lacking the ER membrane complex protein Emc3 or fusion of a C‐terminal hemagglutinin (HA) tag did not erase this background signal (Fig. 2E,F). But when we fused the powerful CL1 degron sequence to the reporter [39], this GFP‐TCS‐quencher‐CL1 (GFP‐Q‐D) sequence showed no background fluorescence (Fig. 2E right panel, F). The CL1 degron sequence consists of 16 residues (ACKNWFSSLSHFVIHL) and is derived from an out‐of‐frame region of the *PMD1* gene. In yeast cells, it facilitates the rapid Doa10‐driven ubiquitination and degradation of improper Pmd1 sequences [42]. Co‐expression of a genetically optimized tobacco etch virus protease (uTEV3, for simplicity here just referred to as TEV) [37] restored a strong fluorescence signal, indicating the proteolytic removal of GFP from the quencher domain (Fig. 2G). Initially, we expressed the GFP‐Q‐D reporter from a single copy CEN plasmid, which resulted in a high cell‐to‐cell heterogeneity (Fig. 2G). However, chromosomal integration of the GFP‐Q‐D reporter resulted in a highly consistent fluorescence signal and was used in the following for all further experiments.

### Fluorescence dequenching can be used to monitor mitochondrial import efficiency

**Fig. 3:**
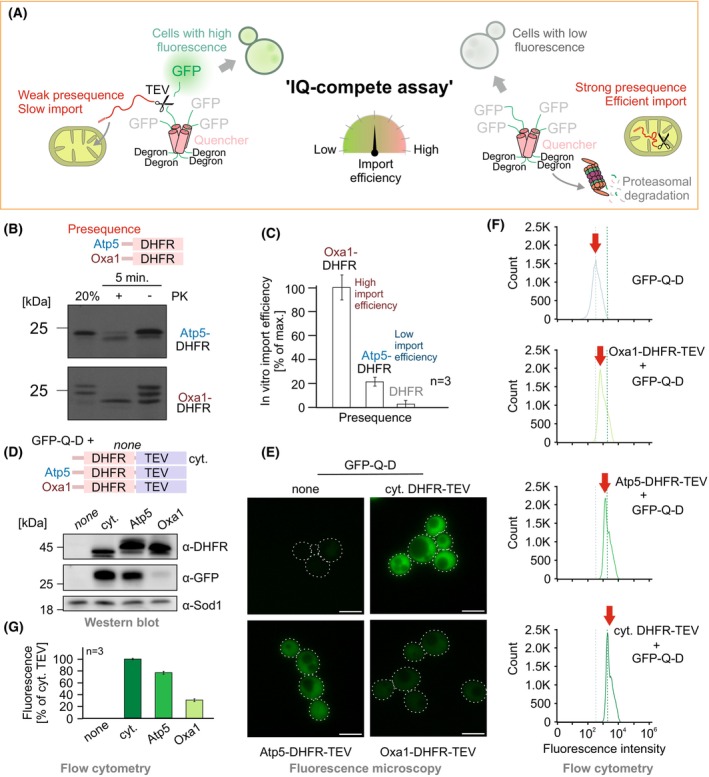
The Import and de‐Quenching Competition (IQ‐compete) assay allows the simple detection of the import efficiency in living yeast cells. (A) Rationale of the IQ‐compete assay. Slow import of the precursor‐TEV fusion releases and thereby activates the fluorescent GFP. On the contrary, efficient import of the fusion protein prevents cleavage and dooms the GFP‐Q‐D reporter to proteasomal degradation. (B) Radiolabeled Atp5‐DHFR and Oxa1‐DHFR were incubated with isolated yeast mitochondria for 5 min. Each sample was split, and one half was treated with proteinase K (PK) to remove the non‐imported protein. The samples were loaded to SDS/PAGE and visualized by autoradiography. The lane on the left shows 20% of the radiolabeled protein that was used per import reaction. DHFR, dihydrofolate reductase. (C) The import experiment shown in B was repeated three times. The signals were quantified. Mean values and standard deviations are shown. For the sample on the right, radiolabeled DHFR (without presequence) was added to mitochondria as a negative control of a non‐imported protein. (D) The GFP‐Q‐D reporter, along with DHFR‐TEV, Atp5‐DHFR‐TEV, or Oxa1‐DHFR‐TEV, was expressed. Cell extracts were analyzed by western blotting. Please note that GFP was only detected in samples in which the cytosolic DHFR‐TEV or Atp5‐DHFR‐TEV construct had proteolytically cleaved the GFP‐Q‐D reporter to prevent its degron‐mediated degradation. cyt., cytosolic. (E–G) The cells used for D were analyzed by fluorescence microscopy (E) and flow cytometry (F, G), always yielding comparable results. In E, scale bars correspond to 5 μm. In F, red arrows indicate the fluorescence intensity corresponding to the maximum number of cells. In G, error bars represent the standard deviation of the mean.

Mitochondrial proteins are imported with different protein‐specific efficiencies, which in part can be attributed to the specific properties of their presequences and their accessibility in a folded precursor protein [16, 30, 43, 44, 45]. To measure the import efficiency of presequence‐containing proteins, we developed the IQ‐compete assay that is based on the GFP‐Q‐D reporter (Fig. 3A).

*In vitro*, fusion proteins consisting of the presequence of Oxa1 and mouse dihydrofolate reductase (DHFR) were very efficiently imported, whereas fusion proteins with the presequence of Atp5 were imported less efficiently (Fig. 3B,C). Therefore, we tested whether the fusion of these proteins to TEV results in cytosolic TEV activity. Western blotting of cell extracts showed that the expression of DHFR‐TEV resulted in a strong GFP signal, representing a GFP‐containing fragment that was released from the GFP‐Q‐D reporter (Fig. 3D). Expression of the Oxa1‐DHFR‐TEV fusion produced only very little of the GFP fragment, indicative of the high import efficiency of the Oxa1 presequence. The Atp5‐DHFR‐TEV fusion protein, which was only inefficiently imported into mitochondria, cleaved the GFP‐Q‐D reporter in the cytosol so that the GFP fragment could be detected by western blotting (Fig. 3D).

This presence of the GFP fragment could be easily measured by western blotting (Fig. 3D), fluorescence microscopy (Fig. 3E), and flow cytometry (Fig. 3F,G). Thus, the quantitative IQ‐compete assay is highly versatile and allows simple detection of the import efficiency in living yeast cells.

### The IQ‐compete assay is robust and independent of protein expression levels and carbon sources

**Fig. 4:**
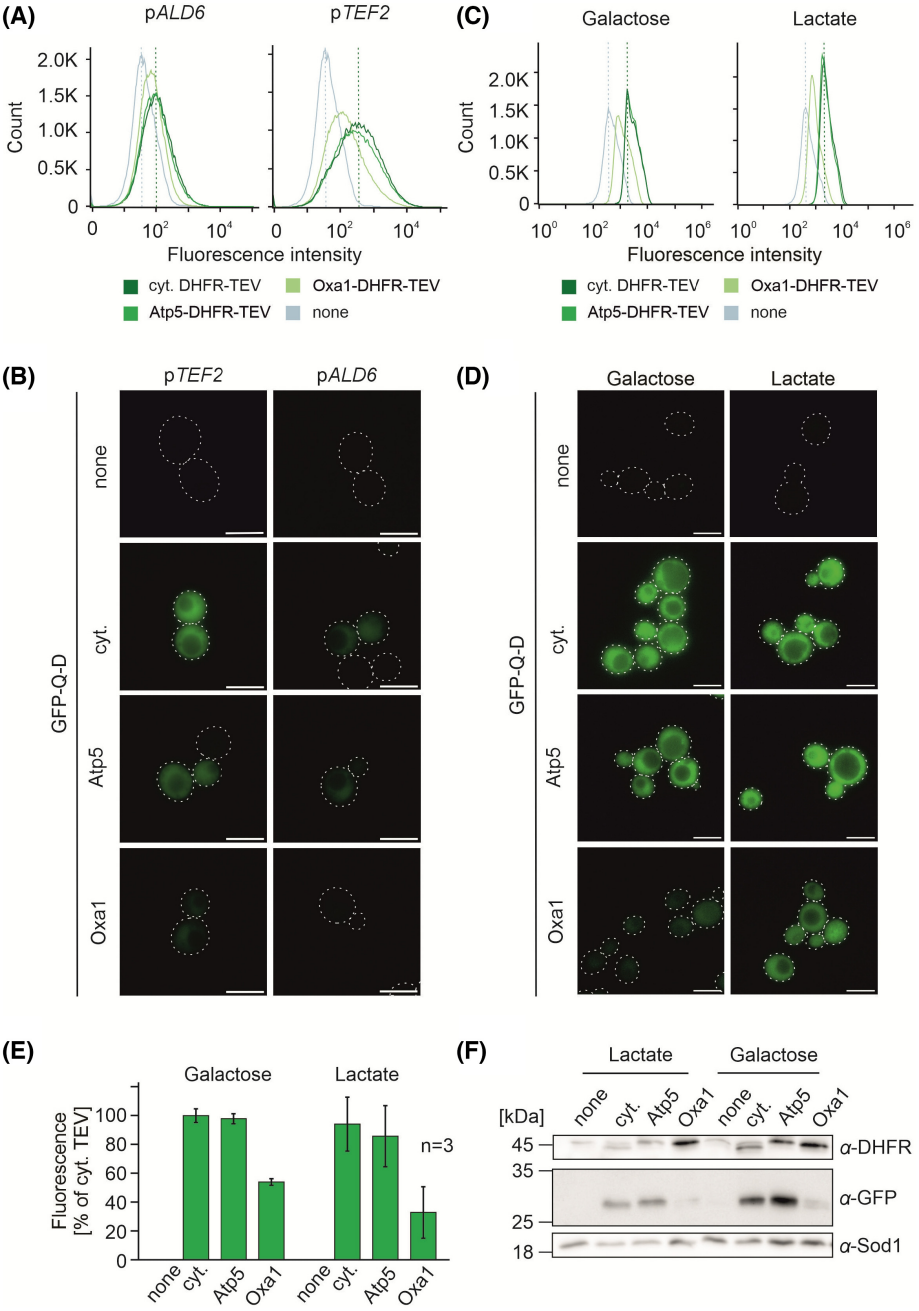
The IQ‐compete assay is largely independent of protein expression levels and carbon sources. (A, B) The indicated DHFR‐TEV fusion proteins were cloned downstream of the moderate *ALD6* or the strong *TEF2* promoter and integrated into the yeast genome before the GFP‐Q‐D‐dependent fluorescence was measured by flow cytometry (A) and microscopy (B). cyt., cytosolic. Scale bars: 5 μm. (C–F) Strains expressing the indicated fusion proteins under control of a *TEF2* promoter were grown to log phase in YP full medium with 2% galactose or lactate, respectively, as carbon sources. The levels of GFP‐Q‐D‐dependent fluorescence and GFP levels were analyzed as described for Fig. 3. In D, scale bars: 5 μm. In E, mean values and standard deviations are shown.

Next, we tested whether the results obtained from the IQ‐compete assay are affected by the expression levels of the reporter. To this end, we expressed the TEV‐containing fusion proteins from a moderate (*ALD6*) or about 3.5 times stronger (*TEF2*) promoter [35] and measured the fluorescence intensities stemming from the cleaved GFP‐Q‐D reporter by flow cytometry (Fig. 4A) and microscopy (Fig. 4B). As expected, the stronger promoter showed higher fluorescence intensities, but the relative differences of the Atp5 and Oxa1 fusions remained constant, showing that the IQ‐compete assay is not affected by the level of expression *per se*.

The mitochondrial volume in yeast cells depends on the carbon sources: glucose represses the synthesis of many mitochondrial proteins, while non‐fermentable carbon sources such as lactate induce respiration and the synthesis of mitochondria [46, 47]. We therefore tested whether the results of the IQ‐compete assay that we obtained on glucose media change upon growth on galactose (fermentation, but no glucose repression) or lactate (respiration). As shown in the Fig. 4C–F, the overall pattern remained identical, indicating that under all these conditions, the presequence of Oxa1 is more efficient than that of Atp5. Thus, the IQ‐compete assay is well suited to monitor the import efficiency of yeast cells grown on different carbon sources.

### The IQ‐compete assay can measure import efficiency at different temperatures

**Fig. 5:**
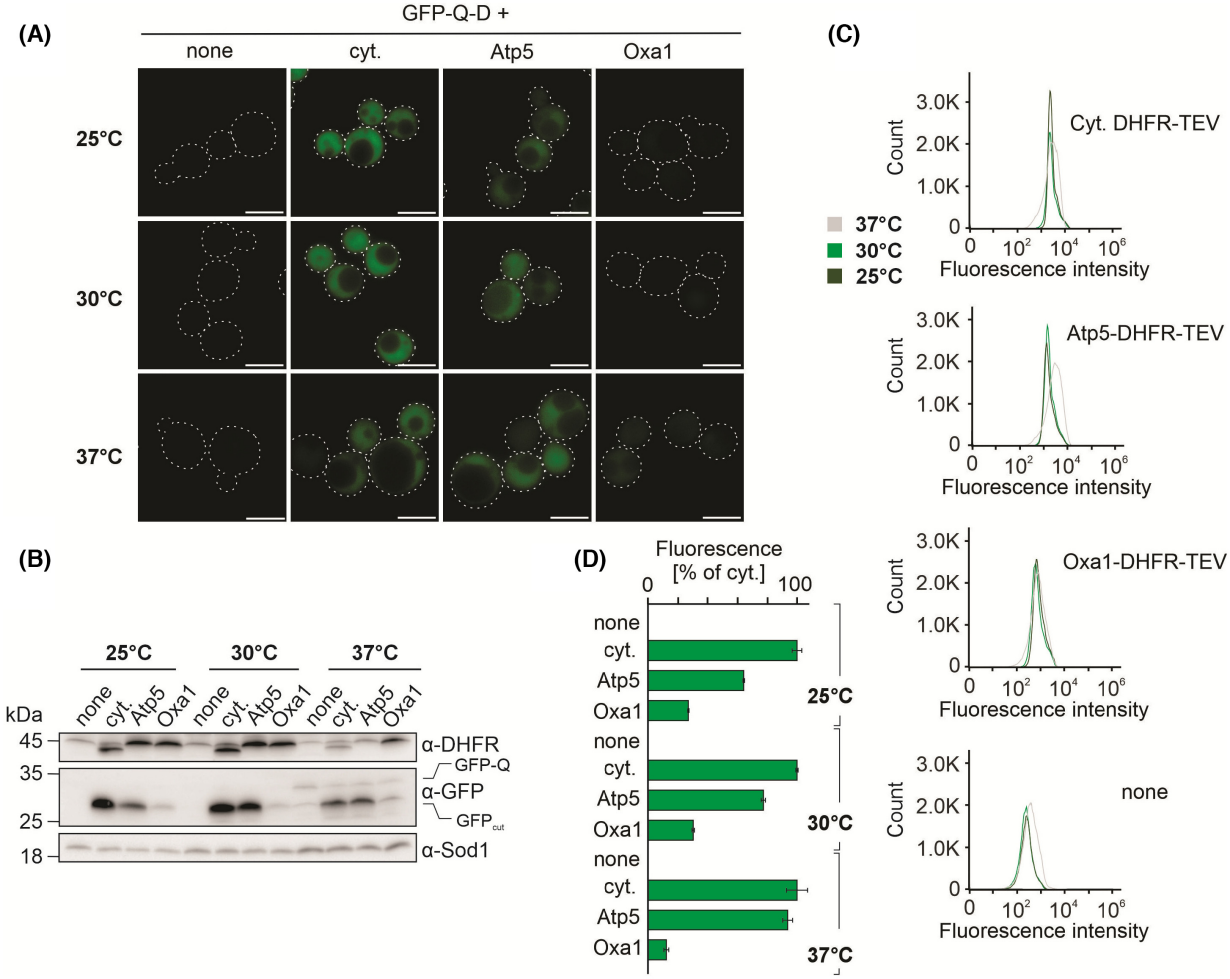
The efficiency of the mitochondrial targeting signals is independent of the temperature. (A–D) Cells expressing the GFP‐Q‐D reporter and the indicated DHFR‐TEV fusion constructs were grown at the indicated temperatures. The efficiency of reporter cleavage was measured by microscopy (A), western blotting (B), and by flow cytometry (C, D). cyt., cytosolic. A, Scale bar = 5 μm. D, Mean values and standard deviations are shown.

Protein synthesis, folding, and import are influenced by the temperature at which cells grow. We therefore tested whether the IQ‐compete assay is compatible with different cultivation temperatures (Fig. 5A–D). We grew cells at 25, 30, and 37 °C and measured the fluorescence signal from the GFP‐Q‐D reporter as described. We found that the incubation temperature had no influence on the assay *per se*, showing that the strong presequence of Oxa1 drives protein import into mitochondria very efficiently under all conditions tested. Thus, the IQ‐compete assay is applicable under different growth conditions.

### The IQ‐compete assay can measure import efficiencies of tagged full‐length proteins

**Fig. 6:**
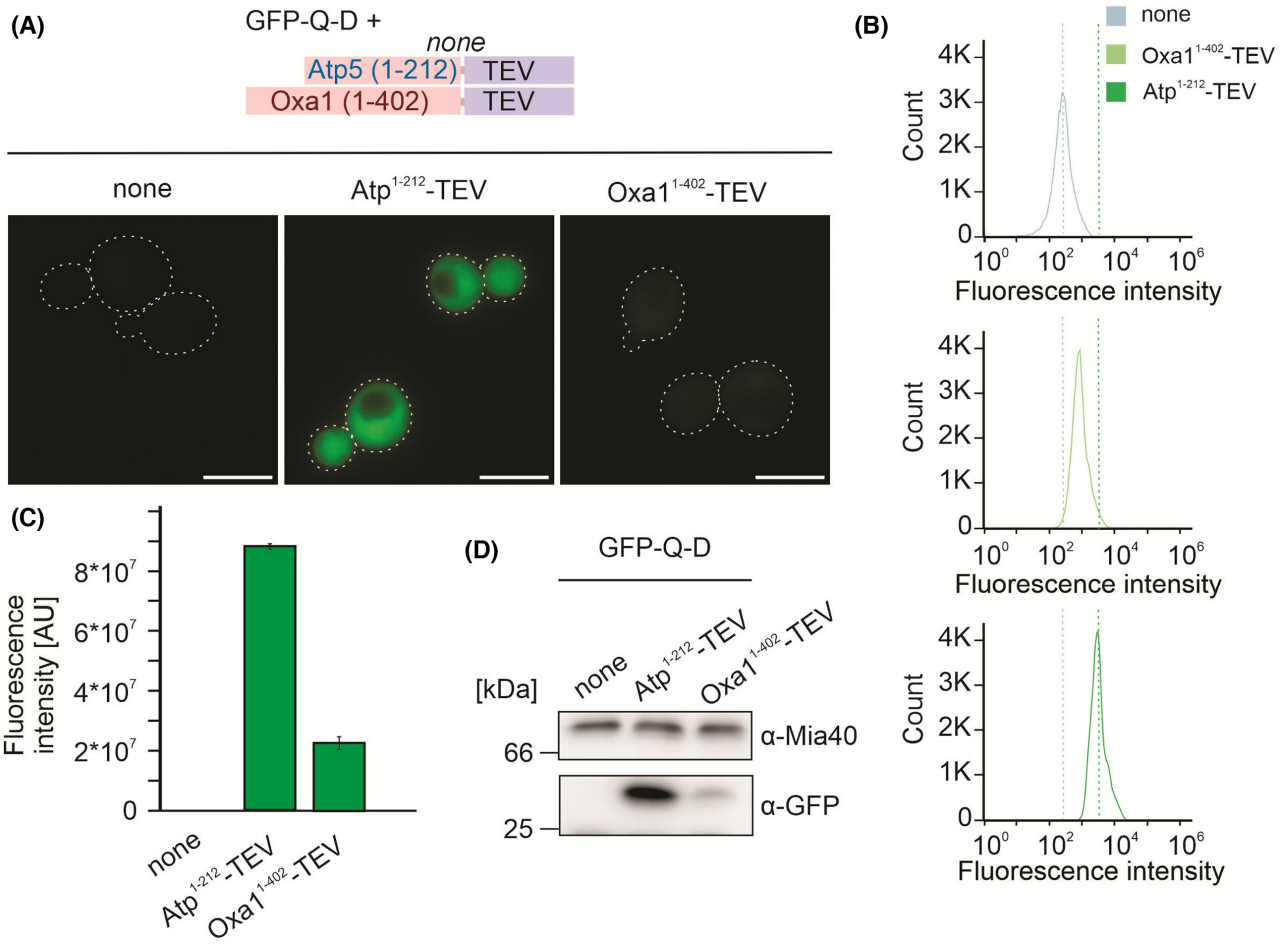
The IQ‐compete assay shows the very efficient mitochondrial import of the Oxa1 precursor. (A–D) Full‐length sequences of the precursor forms of Oxa1 and Atp5 fused to TEV were expressed from a constitutive *TEF2* promoter. Cells were grown in YPD medium to mid‐log phase before samples were analyzed by microscopy (A), flow cytometry (B, C), or by western blotting (D) as described for Fig. 3. A, Scale bars = 5 μm. C, Error bars represent the standard deviation of the mean.

Up to this stage, we have used artificial fusion proteins consisting of mitochondrial presequences and DHFR in order to test the targeting properties of the mitochondrial targeting sequences independent of the mature protein segments. We therefore wondered whether the IQ‐compete assay can also be used to measure the import efficiency of full‐length (native) precursor proteins. To this end, we fused the TEV domain to the C terminus of the precursors of Atp5 and Oxa1 (Fig. 6A–D). We observed that the co‐expression of the GFP‐Q‐D reporter with the Oxa1‐TEV fusion resulted in significantly lower fluorescence signals compared to when the Atp5‐TEV construct was used. This demonstrated that Oxa1 is much more efficiently imported into mitochondria than Atp5. Thus, the IQ‐compete assay offers an efficient and quantitative approach to measure the import efficiency of individual proteins in living yeast cells.

## Discussion

The molecular mechanisms by which proteins are targeted to the mitochondrial surface and threaded through the membranes of mitochondria have been elucidated in great detail [7, 8, 9, 10]. *In vitro* assays with radiolabeled precursor proteins synthesized in reticulocyte extract that were mixed with isolated yeast mitochondria have been the experimental basis of the mitochondrial import community for decades and most of what we know about the features of the import system is derived from this very powerful approach. In this study, we present the IQ‐compete assay as an alternative import assay that monitors the efficiency of the mitochondrial protein import reaction by fluorescence dequenching *in vivo*. In a recent study, we have shown that the import efficiencies of presequences obtained from the IQ‐compete assay correlate very well with the values obtained from the classical *in vitro* assay [16]. The IQ‐compete assay has the distinct advantage in that it can measure import efficiencies in individual cells, so that it is compatible with microscopy or flow cytometry, as shown in this study. Thus, it can be combined with genetic screens for factors that affect the import efficiency or proteolytic stability of mitochondrial precursors or for genome‐wide analyses in synthetic genetic arrays [48].

Several *in vivo* approaches for mitochondrial protein targeting have been published recently (see Fig. 1). A very interesting novel method, the MitoRush system [49] has been described during the revision of this study: For MitoRush, precursor proteins are initially tethered to the ER surface and released in a biotin‐driven chase reaction. Thus, MitoRush is particularly interesting to characterize factors which can promote the unfolding and import of folded precursors. Another interesting method is the split‐GFP assay, which proved to be compatible with genome‐wide analyses [50]. This assay measured the targeting of proteins into the mitochondrial matrix by the complementation of a C‐terminally fused GFP^11^ fragment with a GFP^1‐10^ fragment that was encoded on the mitochondrial genome. This split‐GFP assay is therefore particularly effective for the identification of minor mitochondrial populations of predominantly non‐mitochondrial proteins [50, 51]. The IQ‐compete assay is complementary to this approach because it is particularly well suited to identify cytosolic pools of predominantly mitochondrial proteins. Such a dual localization of proteins was reported to be frequent: up to one third of all mitochondrial proteins were proposed to form minor fractions in the cytosol [52, 53, 54].

Apparently, cells intentionally produce non‐mitochondrial pools of mitochondrial proteins, predominantly for two reasons: First, enzymes can catalyze their metabolic reactions in mitochondria as well as in the cytosol. In yeast, fumarase and aconitase are well‐studied examples that serve in the tricarboxylic acid (TCA) cycle in mitochondria as well as in the glyoxylate shunt in the cytosol [55, 56]. Similarly, several TCA cycle enzymes were identified in the cytosol and nucleoplasm of human cells to produce metabolites required for histone acetylation [57]. In addition, cells use the dual localization of proteins for signaling. The failure to import specific proteins into mitochondria leads to the accumulation of specific precursors in the cytosol which can serve as reporters for mitochondrial dysfunction [58, 59]. These non‐imported precursors regulate cellular physiology on different levels: They increase the activity of the ubiquitin‐proteasome system [59, 60, 61, 62, 63, 64], rewire the cytosolic protein folding network [17, 18, 31, 65, 66, 67, 68, 69], reduce protein synthesis rates [62, 70, 71, 72, 73], induce mitophagy [71, 72, 74, 75], and influence gene expression in the nucleus [62, 76, 77]. The nematode protein Atfs‐1 represents a particularly well‐characterized example of such a signaling molecule: If the mitochondrial membrane potential falls low, Atfs‐1 fails to enter mitochondria but is directed to the nucleus where it triggers a mitochondrial stress‐response program [78, 79, 80].

In this study, we now present a method that reliably detects cytosolic pools of non‐imported mitochondrial precursor proteins. The use of a fluorescence quencher cassette makes this reporter largely independent of proteasome activity. It will be exciting to use this method, for example, to identify cytosolic precursors comprehensively by genome‐wide screens or in combination with genetically encoded redox or metabolite sensors [81, 82, 83, 84]. We are convinced that the IQ‐compete assay described here will be a powerful and versatile novel instrument in the experimental toolbox of the mitochondrial community.

## Author contributions

YH, AE, and SR constructed the strains, performed the biochemical, microscopic, and yeast genetics experiments. YH analyzed and quantified the data. JMH and SR conceived and supervised the study. JMH wrote the manuscript, with all authors critically reviewing it.

## Supporting information


**Data S1.** Original data presented in this article.

## Data Availability

All reagents generated for this study are available upon request from the corresponding author (JMH, hannes.herrmann@biologie.uni-kl.de). The plasmids for expression of Oxa1‐DHFR‐TEV, Atp5‐DHFR‐TEV, DHFR‐TEV, and the GFP‐Q‐D reporter were deposited at Addgene for easy accessibility under the accession numbers #247176 and #247217 – #24720.
