# Supplementary material for: The IQ‐compete assay for measuring mitochondrial protein import efficiencies in living yeast cells
Source: FEBS Lett. 2025 Oct 25;600(1):48–62. doi: 10.1002/1873-3468.70206 (PMC12793721; doi:10.1002/1873-3468.70206)

<u>Raw data</u>

Figure 1

Figure 1G

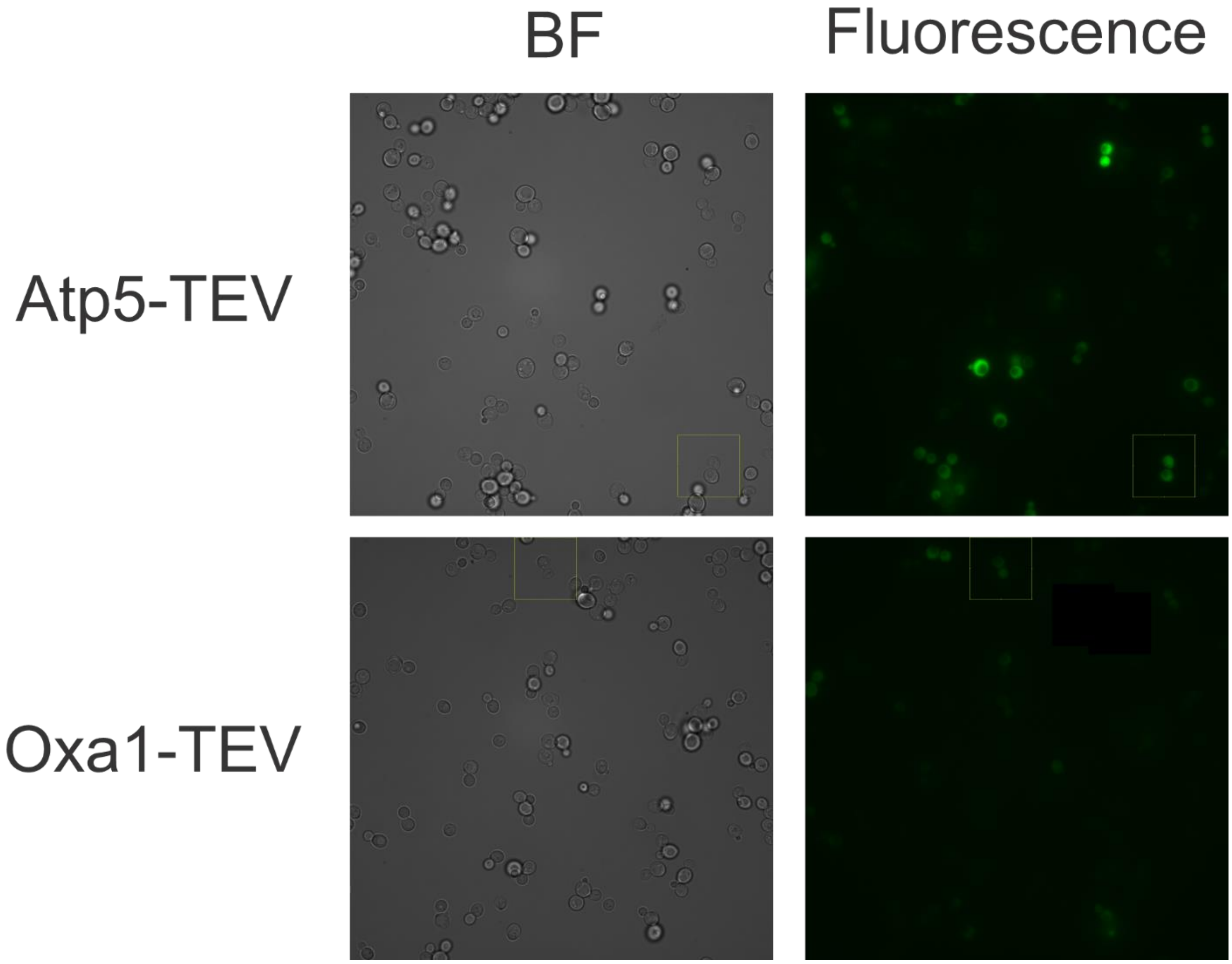

Figure 2

Figure 2E

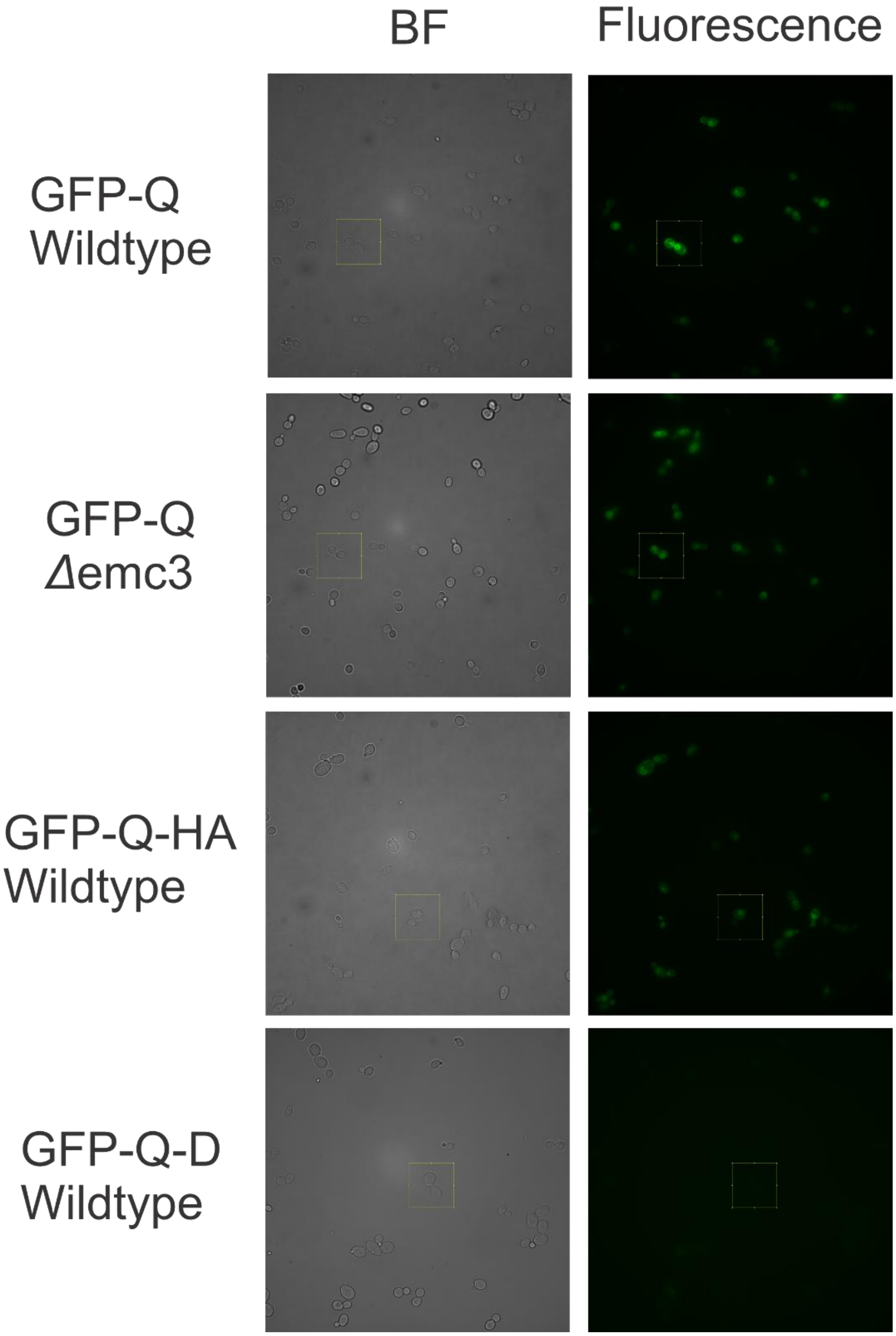

Figure 2G

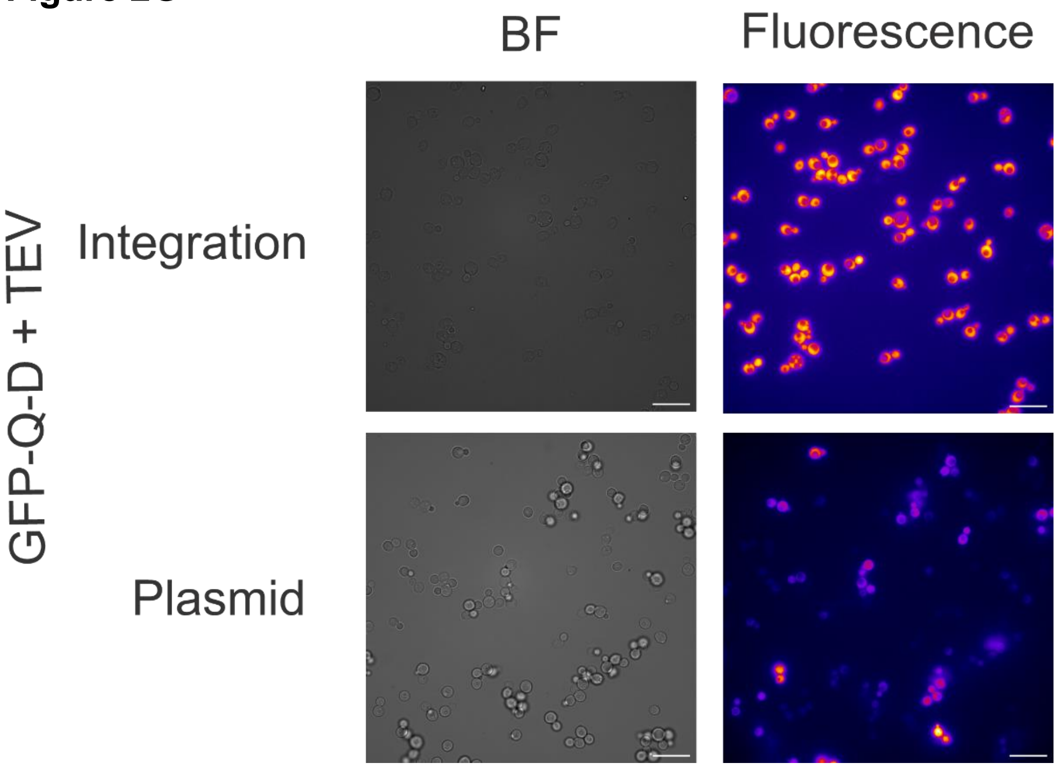

Figure 3

Figure 3B

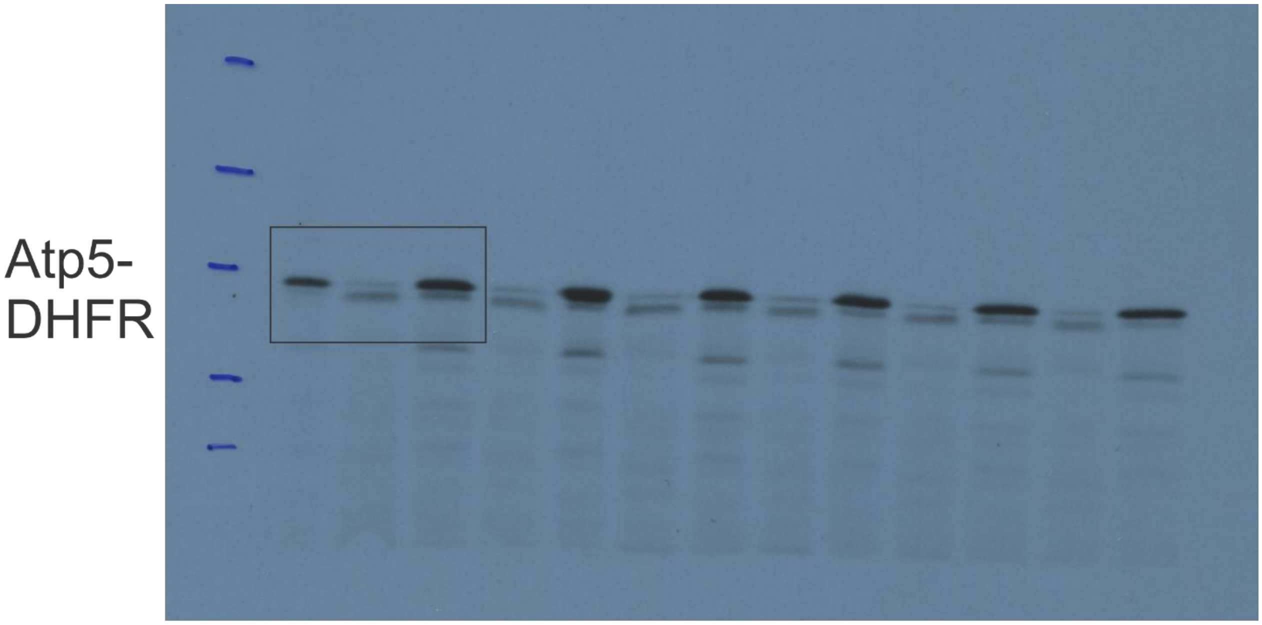

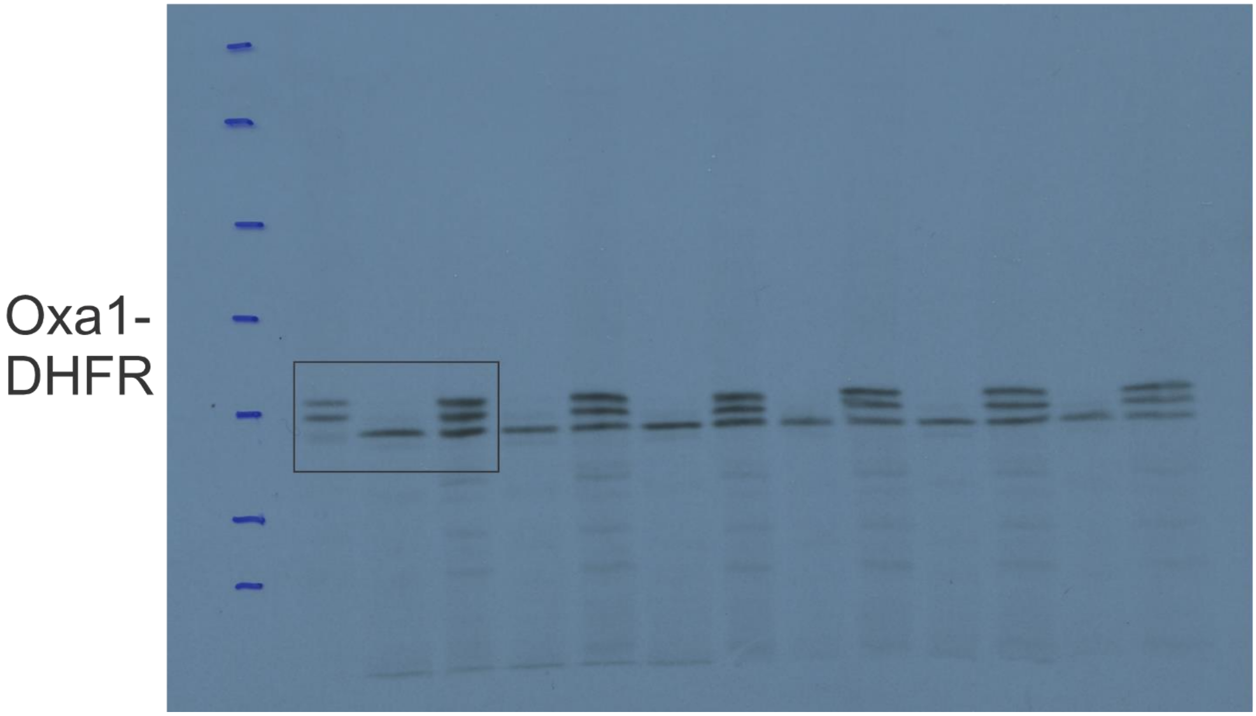

Figure 3D

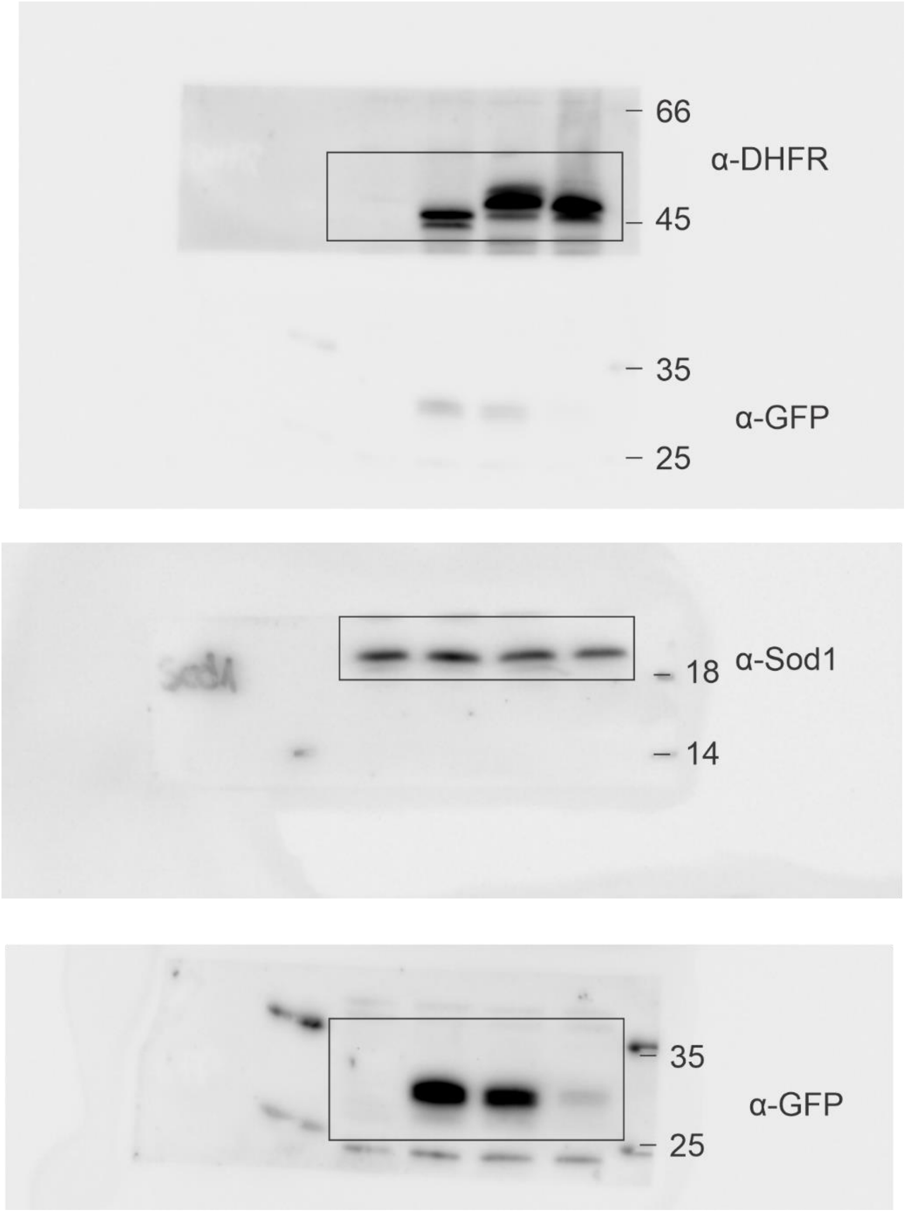

Figure 3F

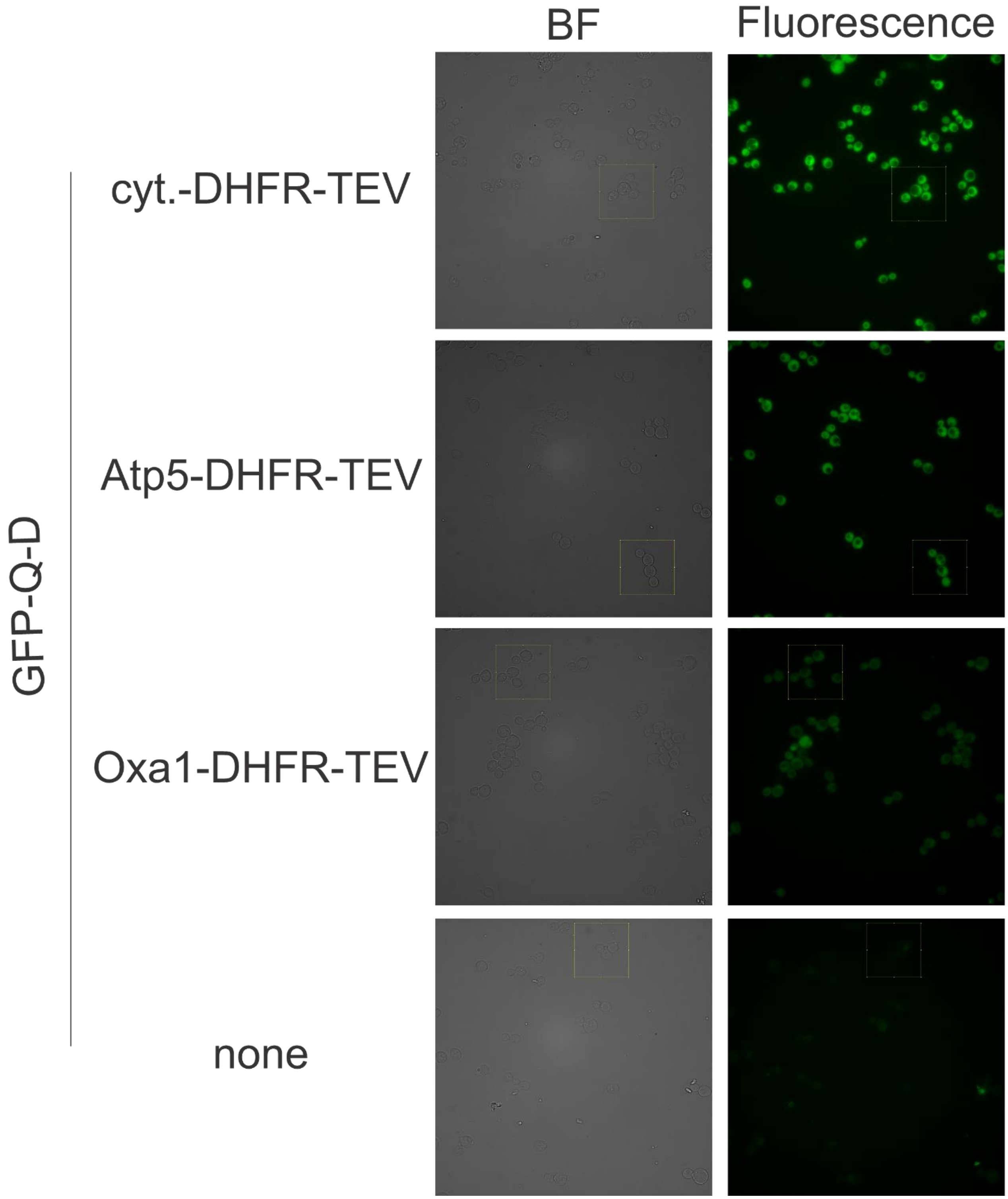
BF
Fluorescence
GFP-Q-D
cyt.-DHFR-TEV
Atp5-DHFR-TEV
Oxa1-DHFR-TEV
none

Figure 4

Figure 4B

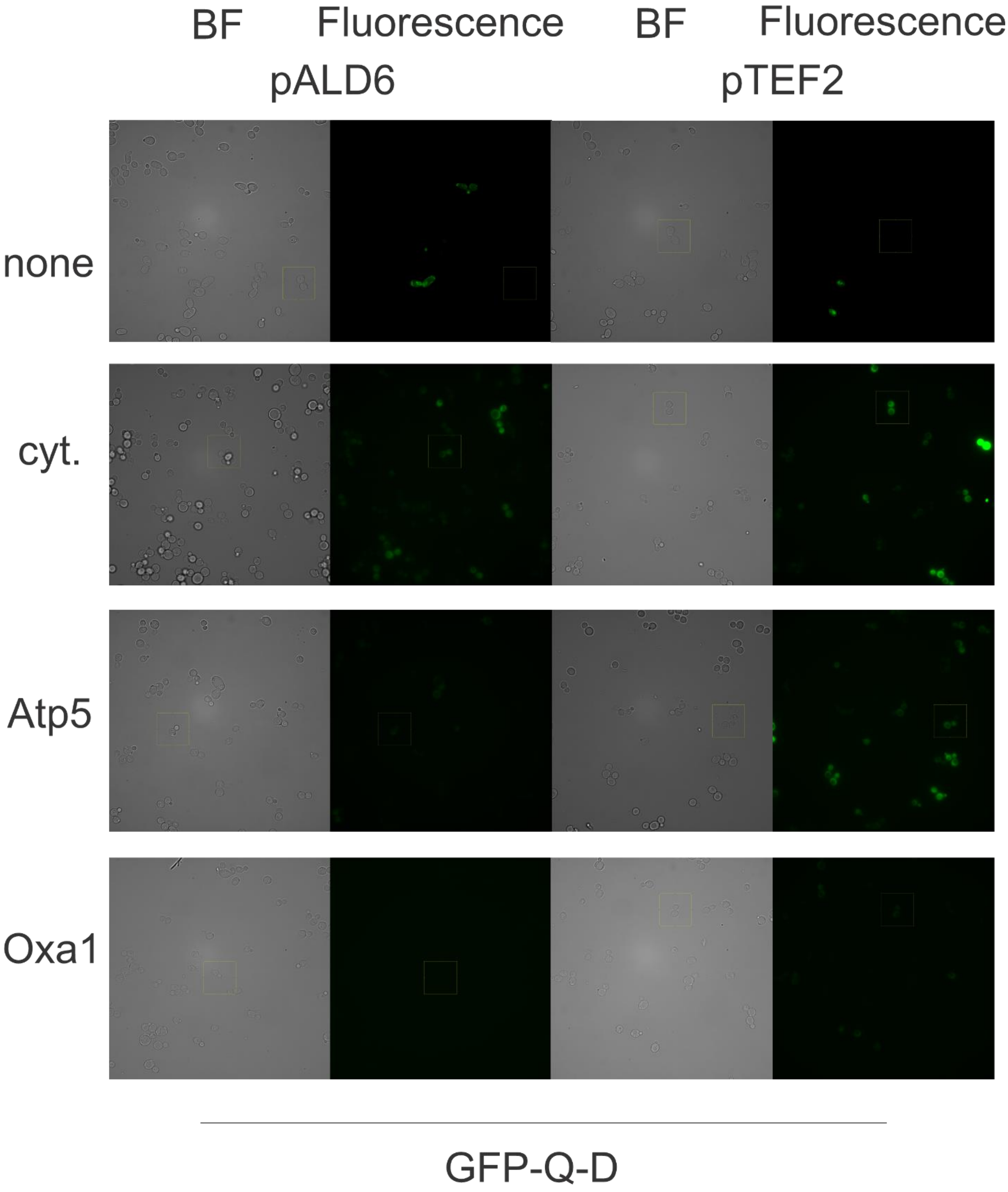

Figure 4D

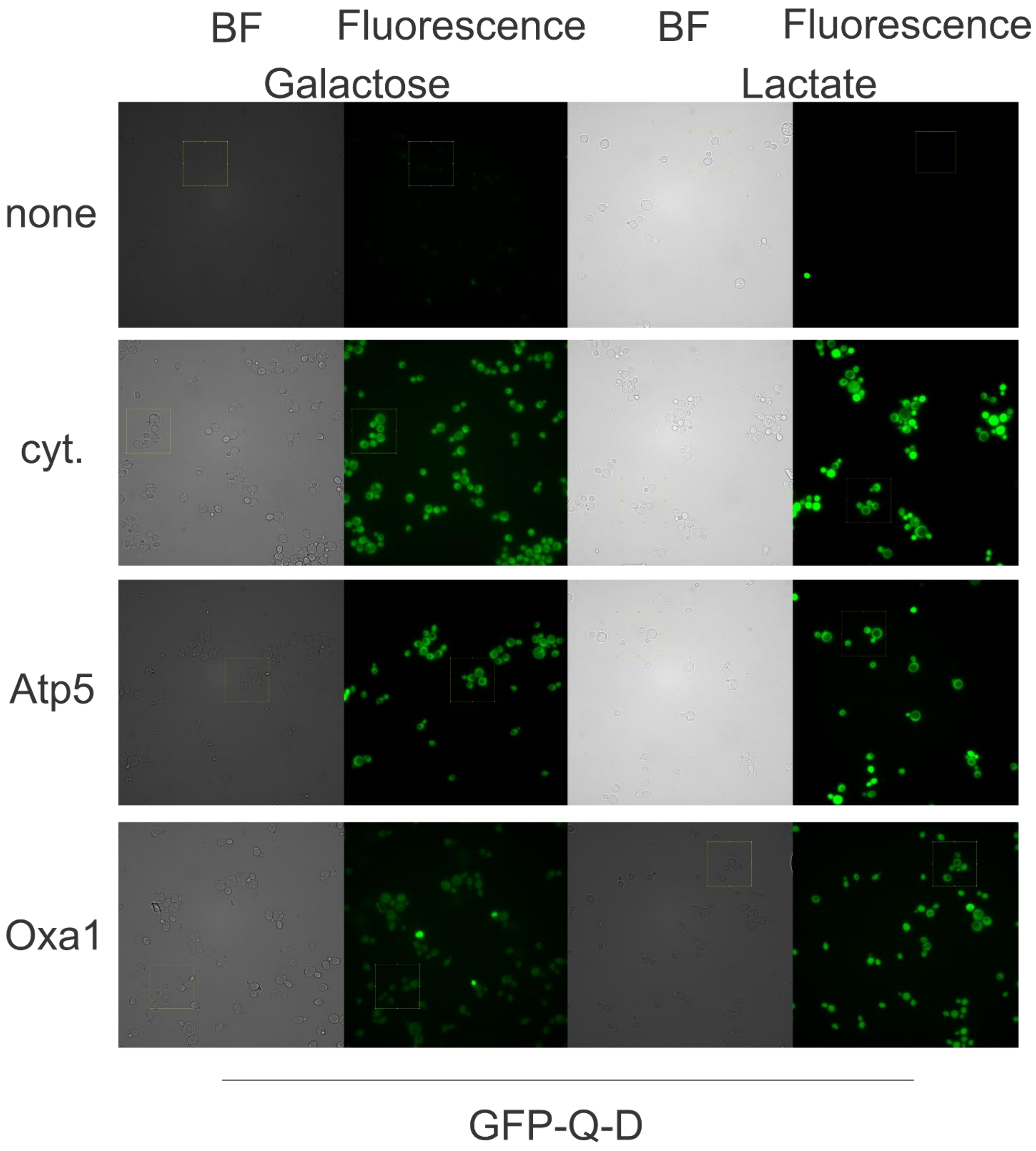
BF
Fluorescence
BF
Fluorescence
Galactose
Lactate
none
cyt.
Atp5
Oxa1
GFP-Q-D

Figure 4F

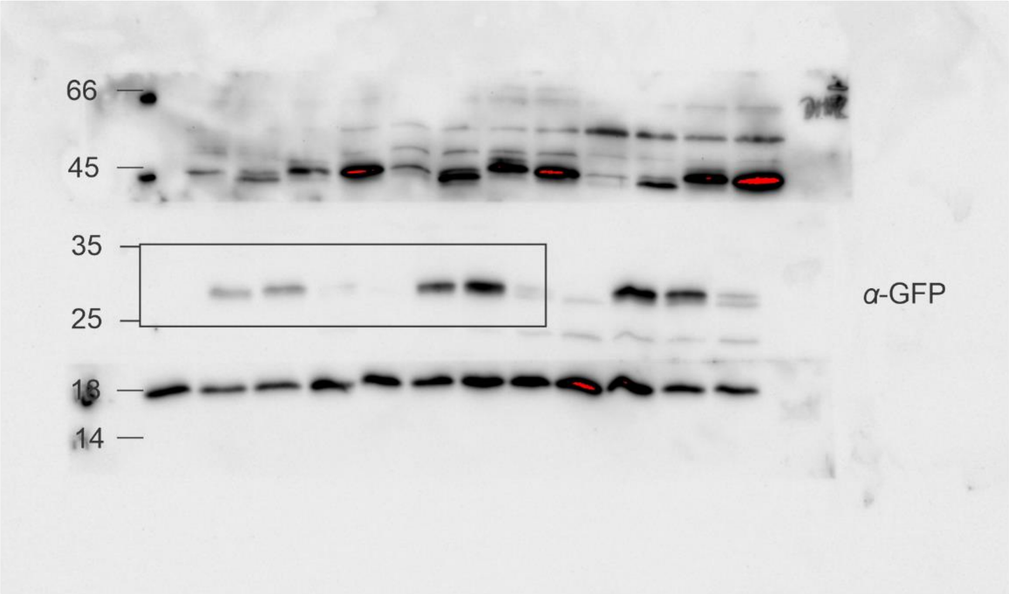
66
45
35
25
18
14
α-GFP

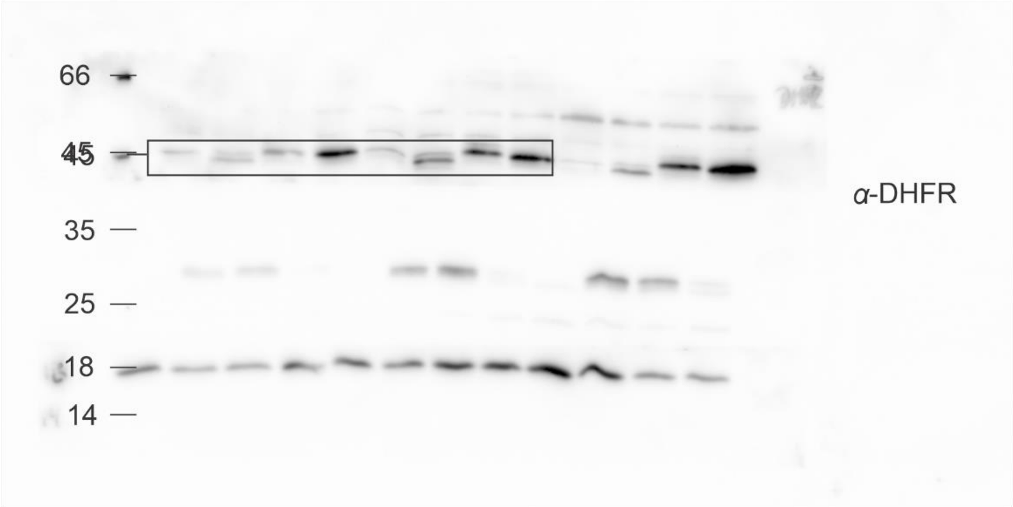
66
45
35
25
18
14
α-DHFR

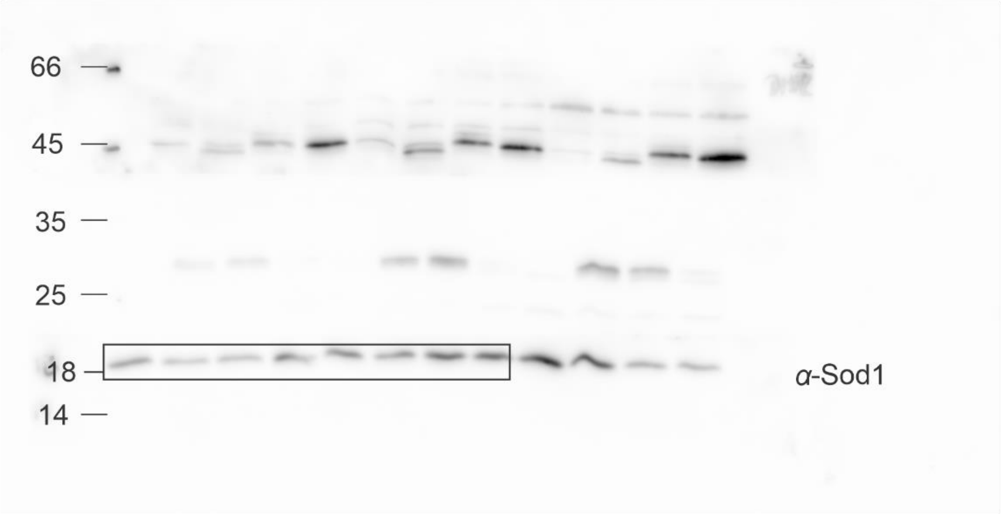
66
45
35
25
18
14
α-Sod1

Figure 5

Figure 5A

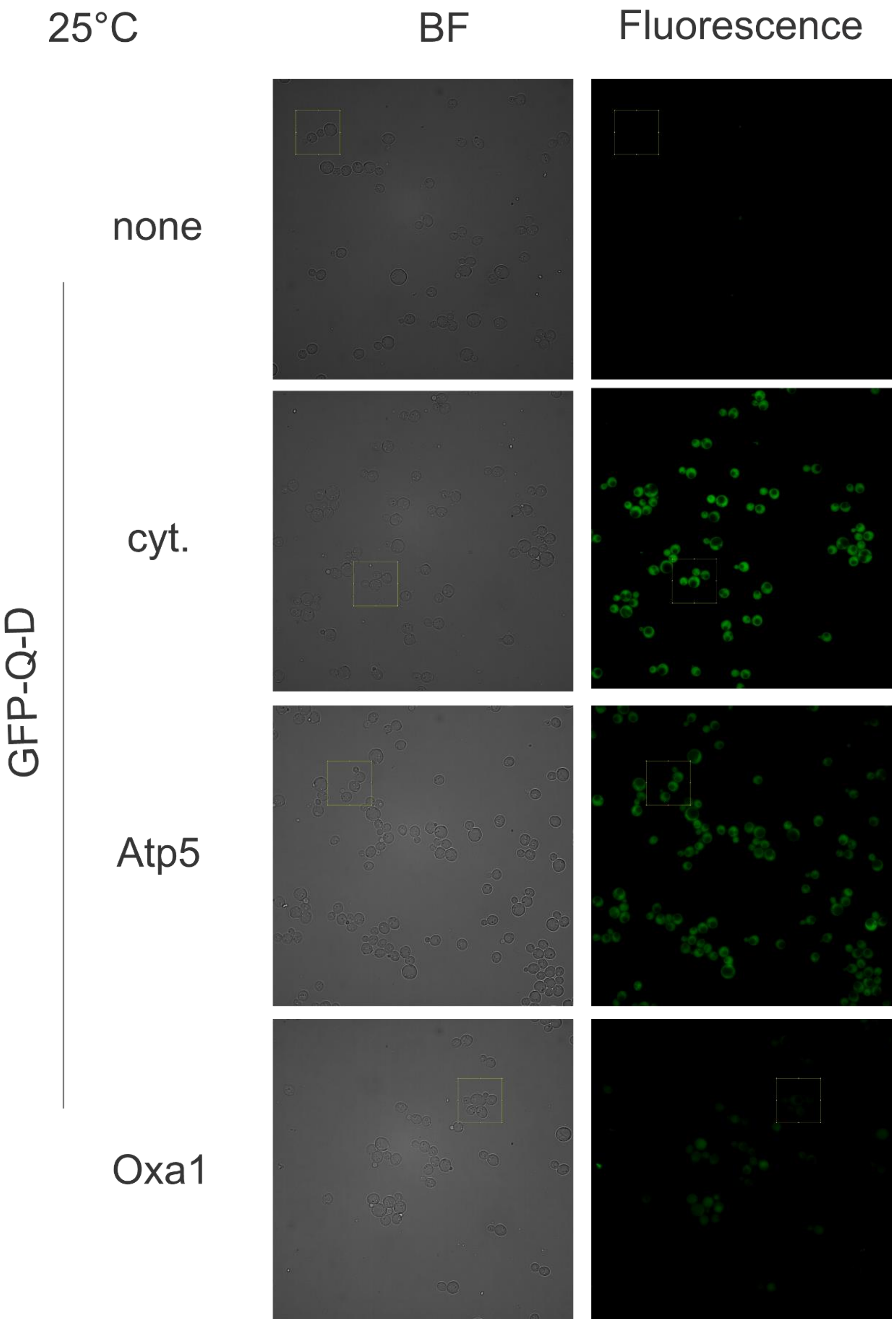

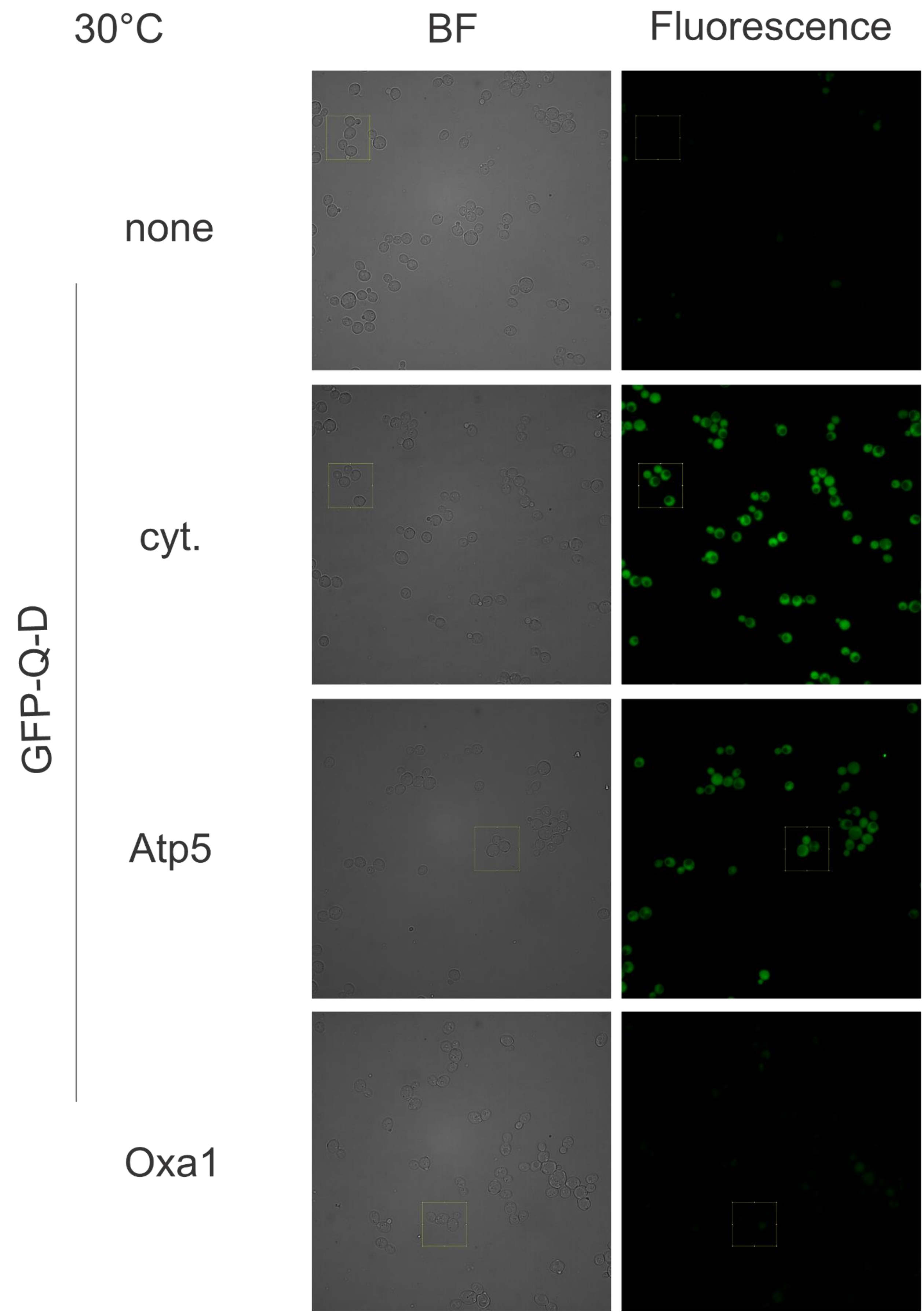
30°C
BF
Fluorescence
none
cyt.
Atp5
Oxa1
GFP-Q-D

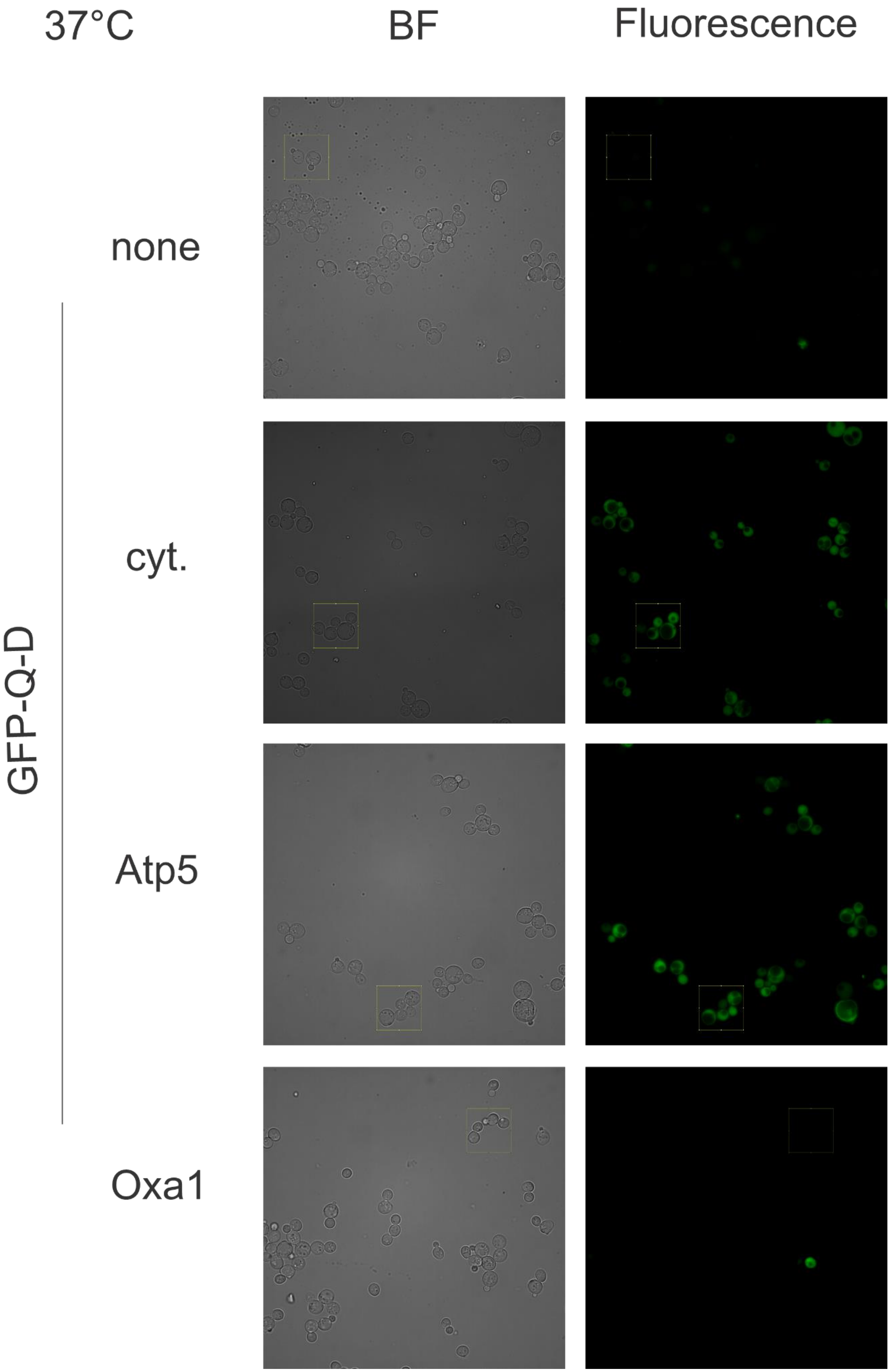

37°C
BF
Fluorescence
none
cyt.
Atp5
Oxa1
GFP-Q-D

Figure 5B

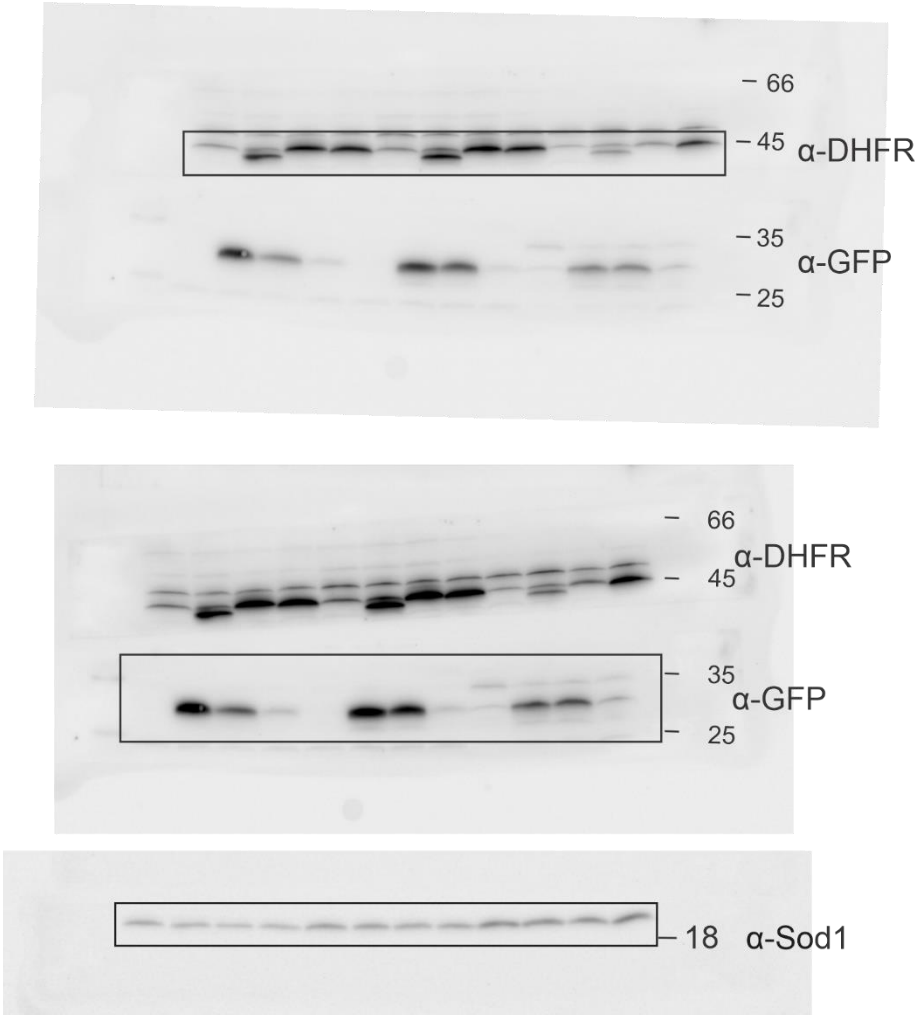

Figure 6

Figure 6A

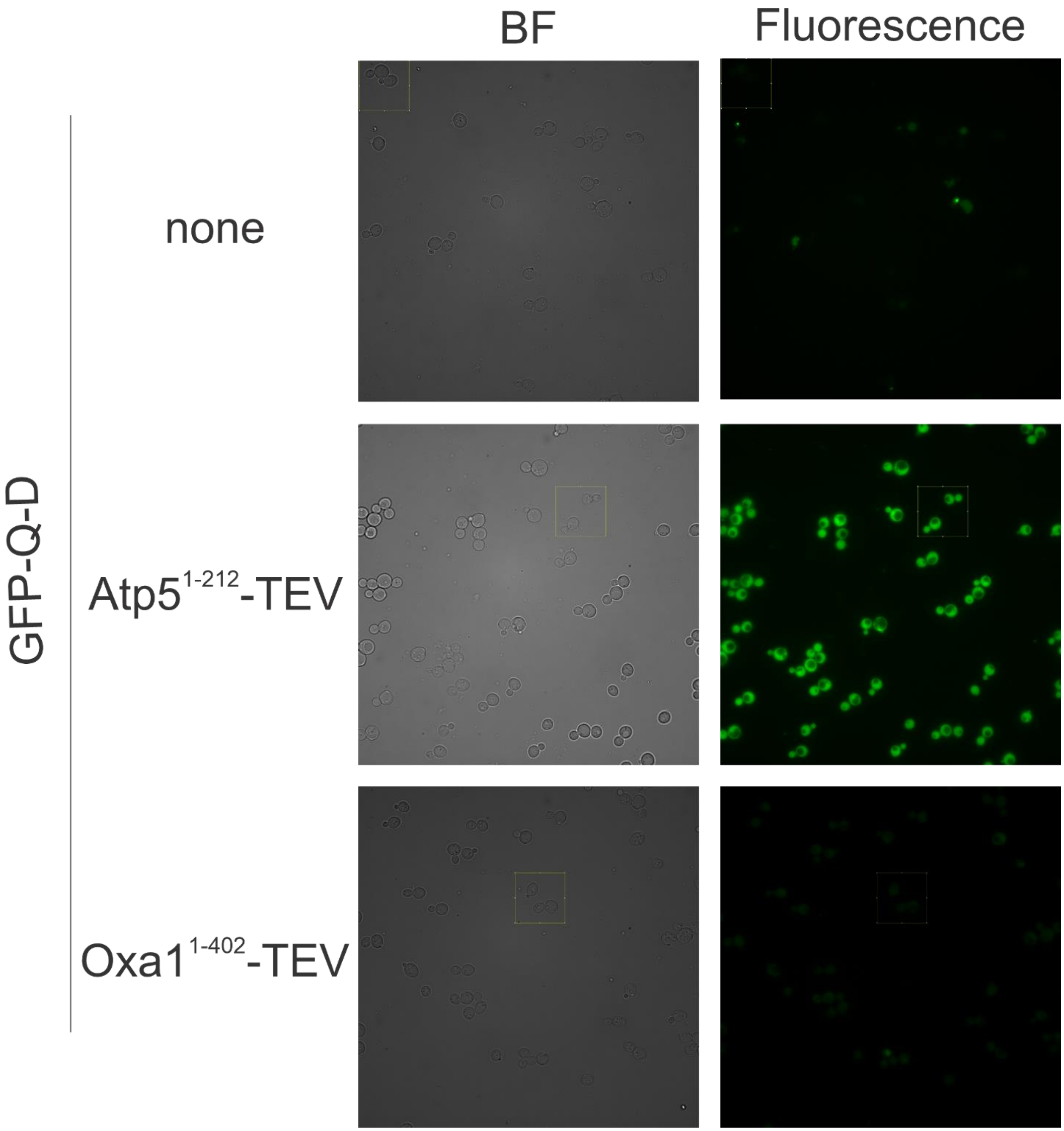

Figure 6D

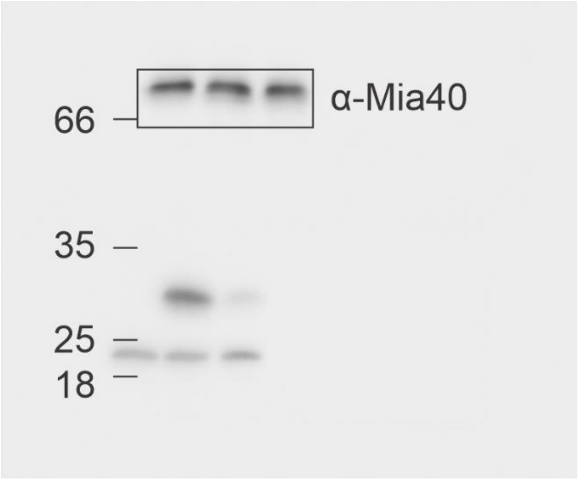

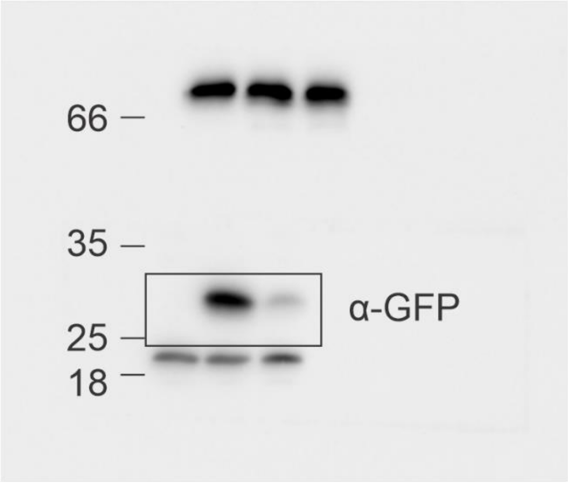

Supplement: Supplementary file 1 — Data S1. Original data presented in this article. [file FEB2-600-48-s001.pdf]
